# Walkability around primary schools and area deprivation across Scotland

**DOI:** 10.1186/s12889-016-2994-0

**Published:** 2016-04-14

**Authors:** Laura Macdonald, Paul McCrorie, Natalie Nicholls, Anne Ellaway

**Affiliations:** CSO/MRC Social and Public Health Sciences Unit, University of Glasgow, 200 Renfield Street, Glasgow, G2 3QB UK

**Keywords:** Children, Schools, Deprivation, Walkability, GIS, Dwelling density, Street connectivity

## Abstract

**Background:**

A number of studies based in the US, Canada, and Australia, have found evidence of associations between the built environment (BE) and mode of transport to school, and links between active travel and deprivation. Limited research in the UK compares potential BE supports for walking to school by area deprivation. Within this study, we gathered data on BE attributes previously linked to active travel, i.e., street/path connectivity, and dwelling density, created a composite ‘walkability score’ (WS) for areas around primary schools across urban Scotland, and explored whether poorer areas exhibit lower scores than more affluent areas, or vice versa. We consider this to be a novel approach as few studies have compared BE features by deprivation across a whole country.

**Methods:**

Address and road/path maps were obtained and primary schools (*N* = 937) across mainland Scotland were mapped. Schools were attributed income deprivation scores (scores divided into quintiles (Q1: least deprived, Q5: most deprived)). Catchment area (CA) boundaries, i.e., the geographic area representing eligibility for local school attendance, were drawn around schools, and WS calculated for each CA. We compared mean WS by income quintile (ANOVA), for all local authorities (LAs) combined (*N* = 29), and separately for the four LAs with the greatest number of schools included in the analysis.

**Results:**

For all LAs combined, the least deprived quintile (Q1) showed a significantly lower WS (−0.61), than quintiles 3, 4 and 5 (Q2: −0.04 (non-sig), Q3: 0.38, Q4: 0.09, Q5: 0.18); while for Glasgow the second least deprived quintile (Q2) showed significantly higher WS (Q1: 1.35, Q2: 1.73), than middling (Q3: 0.18) and most deprived quintiles (Q4: 0.06, Q5: −0.10).

**Conclusion:**

WS differ by deprivation with patterns varying depending on the spatial scale of the analysis. It is essential that less walkable areas are provided with the resources to improve opportunities to engage in active travel.

## Background

A growing body of literature highlights the importance of the built environment (BE) in supporting physical activity (PA), and in particular the importance of ‘walkable’ neighbourhood design or ‘walkability’ in encouraging walking and active transport [1–5]. The term ‘walkability’ has been used to conceptualise a combination of BE factors such as street connectivity, dwelling (or residential) density, net area retail and land use mix; these features have been linked to walking in adults [6, 7], but associations between walkability and active transport for children is less studied and understood [8]. Few studies have looked at how BE features of the neighbourhoods around schools influence active travel to school [9], and any studies conducted to date tend to be based in particular contexts such as the US, Canada and Australia [2, 9–14]. Existing research suggests that walking to school rates are higher when the neighbourhoods around schools have a higher population [10] or residential density [2, 12] (or greater residential density where the distance to school was further than 1 mile [13]), and higher street connectivity (i.e., high intersection density) [10], while other research suggests that when high street connectivity was combined with high traffic volume around schools walking to school rates were reduced [9]. A small number of studies have compared potential BE measures of walkability between schools in poorer or more affluent areas [9, 15], and these studies were limited to exploring walkability within cities. Results have been varied, with some studies showing deprived areas to be less walkable than higher income areas [5, 9], and other research showing deprived areas to be more walkable than their higher income counterparts [15, 16]. There is a dearth of research in the UK comparing potential BE supports for walking around schools by area deprivation. One survey explored walking to school rates by deprivation and found that rates of active travel to schools in Scotland differed by deprivation but the pattern was non-linear; rates of walking were highest in the most deprived areas, decrease with decreasing deprivation, but rise again for the most affluent areas [17, 18]. It could be argued that lower car access in more deprived areas is the main reason for higher walking to school rates, but other factors may be at play as more affluent areas show both high car access and high rates of children walking to school. Previous work has shown the potential for features of the BE to influence mode of transport to school, and area deprivation differences in active transport rates, thus within this study our aim was to examine whether selected features of the BE, i.e., dwelling density and street/path connectivity, differ by degree of affluence. We explore this for primary school catchment areas across Urban Scotland as a whole, and at local authority (LA) level.

## Methods

### Linking schools to deprivation scores

A list of the addresses for all primary schools (i.e., elementary schools educating children aged 4–11 years old approximately) across mainland Scotland was created using Scottish Schools online [19]. Ordnance Survey (OS) Maps, including point addresses and the road and path networks (i.e., motorways, main and minor roads, local streets, private roads (public or restricted access), alleys and pedestrianised streets, and pedestrian paths) [20] were obtained. GIS mapping software (ArcMap v10) was used to geocode the location of each school by the population centroid of their unit postal code (unit postcodes are the smallest level of postal geography in the UK and typically contain around 15 address points). School postcodes were linked to the small-area statistical geography ‘data zones’ [21] and then to Scottish Index of Multiple Deprivation (SIMD) 2012 Income sub-domain scores (scores based on numbers of claimants for a range of welfare benefits e.g., Income Support, Jobseekers Allowance, Tax Credits etc. [22]). We chose not to use the full SIMD as it includes drive time to schools (within the ‘Access’ sub-domain); associating walkability with a measure that included the ‘Access’ domain would be tautological [23]. SIMD scores were divided into quintiles (Q1 = least deprived, Q5 = most deprived).

### Linking schools to Urban/Rural categories

School postcodes were linked to the six fold ‘Urban Rural Classification 2011/2012’ [24] and only those schools within ‘Other Urban’ areas (i.e., settlements of 10,000 to 125,000 people) or ‘Large Urban’ areas (i.e., settlements of over 125,000 people) were selected for inclusion in the analysis. Schools from small towns and rural areas were excluded as their associated catchment areas (CAs) (i.e., the geographic area from which children are eligible to attend a local school) cover much larger areas. The geographic reach of the majority of these schools extend beyond what might be considered as a ‘walkable’ distance between home and school for children, which we suggest could be classified as beyond 2 miles, as this is the threshold at which most LAs provide free bus transport [25]. Only primary schools were included in the study as secondary schools are fewer in number and on average located further from children’s homes. Every mainland LA within Scotland was contacted to request boundary data for primary school CAs; 29 LAs provided data appropriate for use within the mapping software (ArcMap v10).

### Collation of spatial data for the WS

When considering variables potentially linked to walking to combine in the WS, the four component walkability index (comprising street connectivity, dwelling density, land use and net area retail) method [6, 26] has typically been used. We propose that possible BE supports for walking to school should not necessarily include land use or retail data as these factors relate to whether residents in an area have a mixed number of daily destinations to walk to (e.g., local food shops, cafes, shopping centres, etc.), while we are interested in travel to schools only; one previous study found that links between the BE and walking to school were stronger when walkability measures included network features rather than land use or retail variables [27]. Existing work used connectivity and dwelling density (amongst other variables) to explore correlates of active travel to school [9, 10, 28], thus in this study, we include street/path connectivity (i.e., intersections density) and dwellings density in a two component WS (see Table 1 for more details). We use our composite score as a proxy for BE supports for active transport in the absence of walking to school data (and BE features linked to actual walking rates); previous work has used one or more chosen BE features to represent neighbourhood ‘walkability’ in the absence of walking data [15, 29]. A street network dataset and a path network dataset for Scotland (both relating to 2011) [30] were obtained from Ordnance Survey, and for dwelling density a count of the number of dwellings, and land area in hectares, for each data zone (for 2012) was acquired from Scottish Neighbourhood Statistics [31].

**Table 1:** Walkability score components

Built environment feature	Implied relationship with active transport
Dwelling density (i.e., the ratio of residential units to the land area)	With high dwelling densities, areas tend to become less car dependent (e.g., it is more difficult to drive and park) and more convenient for walking.
Street/path connectivity (i.e., the ratio of true intersections to the land area)	When intersection densities are high, the route between origin and destination is more direct and quicker.

### Calculating the WS and linking to school catchments

ArcMap was used to combine the street network dataset with the path network via respective nodes, and for each data zone a measure of street/path connectivity was calculated using intersection density, i.e., the ratio of the number of true intersections (three or more legs) to the data zone area [6]. Z-scores were computed using IBM SPSS Statistics V.21 for both variables to standardise scores, and the following formula was used: *WS = (2 x intersection z-scores) + (dwelling density z-scores)* (similar formula used in [6]). Street connectivity was weighted by two as previous work highlights the strong influence of this measure on active travel choices [32]. Maps of data zone geographic centroids (i.e., data zone centre points) and CAs were overlaid and where a data zone centroid fell within a CA boundary it was linked to that CA. Mean WS was calculated for data zones within each CA, allowing each school to be allocated a WS.

### Statistical analysis

IBM SPSS Statistics V.21 was used to compare mean WS by income quintile (parametric Analysis of Variance (ANOVA)) across quintiles. Comparisons were made by certain LAs, i.e., the LAs with the greatest number of schools/CAs included in the analysis, i.e., Glasgow: 138 schools, North Lanarkshire: 92 schools, Edinburgh: 80 schools, South Lanarkshire: 74 schools. Post-hoc tests were also carried out to explore differences between quintiles highlighted by ANOVA, using Bonferroni correction to reduce the likelihood of Type 1 errors.

## Results

937 primary schools across 29 LAs were included in the analysis with mean WS ranging from -4.90 to 20.26. There were significant differences *across* the quintiles of income deprivation in mean WS (*p* < 0.001); the least income deprived quintile (Q1) showed the lowest mean WS (−0.61), followed by the second least deprived quintile (Q2) (−0.04), while Q3 (middling) showed the highest mean WS (0.38), followed by the most deprived (Q5) and second most deprived (Q4) quintiles (values of 0.18 and 0.09 respectively) (see Table 2). When comparing WS *between* quintiles, Q1 varied significantly to quintiles 3, 4 and 5 (p-values all less than 0.05), but there were no other significant differences between other quintiles.

**Table 2:** Mean walkability score by income deprivation quintile

Income Quintile	Mean	N	Std. Dev.	Minimum	Maximum
Urban Scotland					
1. (least deprived)	−0.61	187	2.44	−4.19	14.93
2	−0.04	188	2.37	−4.64	7.46
3	0.38	187	2.51	−4.83	9.75
4	0.09	188	2.17	−4.90	7.61
5. (most deprived)	0.18	187	2.50	−3.00	20.26
Total	0.00	937	2.42	-4.90	20.26
ANOVA *p*-value	<0.001				
Glasgow City					
1. (least deprived)	1.35	27	2.66	−3.98	6.07
2	1.73	28	2.41	−1.65	6.57
3	0.18	28	1.59	−1.97	4.81
4	0.06	28	1.38	−2.39	3.48
5. (most deprived)	−0.10	27	1.30	−1.96	2.76
Total	0.64	138	2.06	-3.98	6.57
ANOVA *p*-value	<0.001				

When comparing income quintiles for WS in Glasgow, Edinburgh, North Lanarkshire, and South Lanarkshire, separately, only Glasgow showed significant differences (*p* < 0.001). Within Glasgow the highest mean scores were found for Q2 and Q1, at 1.73 and 1.35 respectively, while the lowest scores were found within the most deprived quintile (−0.10), followed by the second most deprived quintile (0.06), and Q3 (0.18) (see Table 2). Between quintile comparisons showed that Q1 did not vary significantly with any of the other quintiles, but Q2 varied from quintiles 3, 4 and 5 (*p*-values all less than 0.05).

## Discussion

Built environment features within Scotland as a whole, and Glasgow in particular, follow different spatial patterns by income deprivation. When looking at CAs within Scotland overall, dwelling and intersection densities, in a combined WS, were lowest within the most affluent areas around urban primary schools and highest within the middling quintile (Q3). Within one particular LA, Glasgow City, the poorest areas displayed lower WS, while Q2 showed the highest mean scores. Additional analysis (data not shown), comparing deprivation patterns of dwelling density and intersection density separately, showed that dwelling density was highest in Q2 for both Glasgow and Scotland, while connectivity was highest in Glasgow for Q2 and across Scotland in Q3; for Scotland the highest WS in Q3 was therefore driven largely by connectivity.

In terms of results for primary schools across Scotland, our findings correspond to some degree with a previous study (‘Growing up in Scotland’ Study (GUS)) which looked at active travel to primary school by income deprivation quintile across Scotland [17]. Results from GUS found that the highest percentage of children who walked to school was within quintiles 4 and 5 (58 % and 64 % respectively) which is comparable with our findings in that Q4 and Q5 had higher scores than Q1 and Q2 (only Q2 significantly different). However, our study showed that Q3 had the highest WS while findings from GUS found this quintile to have the lowest percentage of walkers (42 %) [17]. It is possible that other factors, not included in our composite score, are impacting BE supports for walking; factors which may be unique to the middling quintile. Similar to our research, a study based in Austin, Texas found that schools in higher income areas appeared to be less walkable for selected BE features, i.e., lower values for students living near to school, sidewalk completeness and population density, however these areas also showed better neighbourhood level safety in terms of lower vehicular crash rates and crime rates (i.e., homicide, rape, robbery, aggravated assault, burglary, larceny theft, arson) [15]. Further work based in Perth, Western Australia, found that even when areas around school were highly walkable in terms of street connectivity, high traffic exposure decreased children’s likelihood of walking to school [9]. Such findings point towards the potential for unfavourable social characteristics of an area, e.g., neighbourhood safety, to offset benefits of the BE for walking [33].

Similarly to our results for Glasgow, the Perth study found areas around lower socio-economic schools to be less walkable, in terms of street and pedestrian connectivity and traffic exposure, than areas around higher SES schools, with findings being driven mainly by lower connectedness in the pedestrian network [9]. Within Glasgow, the second least deprived quintile (Q2) showed the highest densities (separately and in the combined score), this could be due to Q2 neighbourhoods being located within or near to Glasgow city centre, i.e., nearby affluent neighbourhoods in the north west (known locally as the ‘west end’) and south of the River Clyde (the ‘south side’), while Q1 neighbourhoods are more residential [34]. The centre of Glasgow is the central business district, while areas within the west end and south side are busy service retail areas; this could explain higher street connectivity and density observed there, contributing to higher WS in those areas. School neighbourhoods within the most deprived areas, on the other hand, tend to be further from these business and service hubs, and are mainly located within peripheral housing estates, and showed the lowest WS. Such estates were built in the 1950s to meet demand for social housing, and housing strategy dictated that they contained low rise tenement flats, and terraced/semi-detached houses [35] with wide streets and many cul-de-sacs (i.e., streets/paths closed at one end) often present; this type of housing/street design could result in low street connectivity [29]. Other LAs included in the study, such as Edinburgh, North and South Lanarkshire, showed no significant differences in WS by deprivation; this could be associated with different geographical distributions of schools by deprivation which do not lend themselves to such stark contrasts in walkability as within Glasgow. There may be something distinct about the historical nature of housing strategy and urban planning within Glasgow that sets the City apart from other LAs (and Scotland in general), which contributes to the variation in BE features by deprivation.

Our research has a number of limitations. We included schools within urban areas only, and excluded rural schools; as rural CAs tend to cover much larger geographical areas. We found, when initially comparing urban and rural areas, that two-thirds of CAs in rural areas (remote and small towns) extended beyond a ‘walkable distance’ of 2 miles around schools, while around 90 % of CAs in large urban areas are classed as ‘walkable’. It is likely that different factors should be explored when considering walkability in rural environments as walking infrastructure, public transport and commuting distance will differ considerably from urban areas [36]. We recognise that some children do not attend the school in their local CA so will have a greater distance to travel, that may be impractical to walk, however in Scotland these children are in a minority; across the 29 LAs, in 2008/09, around 1 in 5 children beginning primary school had placing requests granted to attend school out with their CA [37] and these are likely to be fairly close. We have no information on distances from home-to-school, a factor previously shown important in the likelihood of walking [11, 38, 39], but an earlier study focussing on Glasgow showed that although, in general, shorter distances to school increased the likelihood of walking, for some schools even with a higher average home-to-school distance for pupils, the prevalence of walking to school remained high [18]. We did not include actual rates of active transport for children in every Scottish primary school within our study as comprehensive and robust data is not readily available. Furthermore, it is beyond the remit of this current study to gather mode of transport to school data for the whole of Scotland, previous work gathering and analysing such data did so for smaller regions (i.e., City wide [9] or areas within a State [10, 13]). We did not gather data on ‘non-built form’ factors such as perceptions of neighbourhood/traffic safety; in some research parental perceptions and concerns have been shown to be a strong predictor of children’s active transport [2], with the likelihood of walking to school increasing with greater perceived neighbourhood or traffic safety [14]. However, in other work no such link between parental traffic safety concerns and active travel were found [40]. Furthermore, we did not explore environmental factors that could contribute to parental fears for their children’s ‘safety’ during active travel. As discussed earlier, previous work demonstrated variation in safety factors by area deprivation levels, such as crime [15], and traffic levels [9, 41], and the potential for these factors to interact with the BE and reduce the walkability of an area. The addition of such factors was out with the scope of this study but could be included in future work where this data could be combined with children’s exact routes. We are currently collecting this type of data using GPS devices within the SPACES Study [42], and aim to include additional attributes in a combined walkability score in future research when such data is more readily available.

Despite the limitations of this research, we believe this study to be of value as there appears to be scarce literature comparing BE features around schools by socio-economic measures such as area deprivation. Our study not only includes analysis at a national level but also at a more local level i.e., LAs; the variation in findings between urban Scotland in general, and Glasgow City specifically, highlight the importance of analyses at different geographical scales. Furthermore, we made use of robust objective measures to encapsulate walkability, using a combination of two key attributes, i.e., street/path connectivity and dwelling density, within GIS, shown to be effective in measuring walkability [32]. Previous research has made use of street networks only and not incorporated less formal walking routes, such as paths, which could lead to underestimation of walkability in some neighbourhoods [43]. In our study we acknowledge that both street and path networks would be used by pedestrians therefore both should be included in any objective measure of walkability. A further strength in our method was the use of CAs rather than buffers around schools, as CAs include the areas around the school, and the home, and to a certain degree the route between both; areas which are important to include when considering potential influences on walking to school [44].

Research exploring potential inequalities in the BE supports for active travel to school is particularly valuable within the context of Scotland as over a quarter of Scottish children do not meet government recommended physical activity levels; with lower levels observed among girls, and decreasing levels as children age [45]. Active transport, provides an opportunity for increased physical activity while undertaking purposeful journeys, such as school travel [46], it is simple, familiar, inexpensive, generally accessible [5, 47], requires no expert skills or equipment [48], and can provide children with social benefits such as ‘walking and talking’ with friends on the route to school [48]. Furthermore, children who regularly walk to school have greater overall activity levels than those who are driven by car [49, 50]. Despite many benefits, only around 50 % of children from urban areas in Scotland regularly walk to and from their primary school [17], and these rates have remained stable in the last five years [51]. In terms of future recommendations, it is clear from the variation in country-wide and regional findings, within this study, that strategy to improve neighbourhood supports for active transport to school should focus on smaller geographical areas, such as neighbourhoods with varying social disadvantage within LAs. Those involved in developing LA urban and transport policies should work towards providing improved street connectivity and reduced levels of traffic on school routes, and ensure CAs do not extend over areas far from schools [9]. A greater number of schools and children could be involved in ‘walk-to-school’ days or weeks, organised by charities such as Living Streets; which aims to create pedestrian friendly streets that are both safe and attractive, and campaign e.g., for safer routes to school [52]. These activities could enhance the sociability of walking to school, a factor which children themselves consider key in promoting the benefits of active transport [48]. Furthermore, improvements to the physical environment must be combined with strategies to involve parents in encouraging children to walk to school, while feeling confident that their children will be safe doing so.

## Conclusion

Our study of urban areas across Scotland contributes to the limited research on potential variations in selected BE features, potentially associated with walking, around primary schools, by income deprivation. Disparities in composite walkability scores, according to deprivation, emphasize the need for specific areas to be allocated resources to improve opportunities for active transport, while the difference in findings between mainland urban Scotland and Glasgow City highlight the importance of exploring and comparing findings at different geographical scales, whether national or city wide.

### Availability of data and materials

We welcome proposals for collaborative projects and data sharing. The raw data in excel file format could be provided via email by the corresponding author upon request for research purposes only.

## Abbreviations

ANOVA: analysis-of-variance
BE: built environment
CA: catchment area
GIS: geographic information system
LA: local authority
OS: ordnance survey
PA: physical activity
Q: quintile
SIMD: scottish index of multiple deprivation
WS: walkability scores
